# Exploring hybrid models for identifying locations for active mobility pathways using real-time spatial Delphi and GANs

**DOI:** 10.1186/s12544-024-00685-7

**Published:** 2024-11-07

**Authors:** Yuri Calleo, Nadia Giuffrida, Francesco Pilla

**Affiliations:** 1 University College Dublin, Dublin, Ireland; 2 Polytechnic University of Bari, Bari, Italy

**Keywords:** Spatial planning, Real-time spatial Delphi, Artificial intelligence, Generative adversarial networks

## Abstract

The spatial planning process is considered an extremely complex system, as it comprises different variables that interrelate and interact with each other. Effectively addressing this spatial complexity necessitates a multidisciplinary approach, as unified methodologies may prove insufficient. Specifically, in urban planning, it is increasingly crucial to prioritize bike lanes, bike stations, and pedestrian zones, for functional transportation infrastructures. This approach can enhance cities by improving air quality, reducing emissions, and boosting public health and safety through physical activity and accident prevention. However, implementing these changes requires careful planning, community engagement, and stakeholder collaboration. This paper proposes a hybrid model for identifying optimal locations for bike lanes, bike stations, and pedestrian zones adopting Real-Time Spatial Delphi and Generative Adversarial Networks (GANs). The Real-Time Spatial Delphi is a modified version of the traditional Delphi method that incorporates real-time feedback and visualization of group response in real-time, aiming to achieve a convergence of opinions among experts on the territory. Nevertheless, these judgments are a spatial representation not visible in reality, and with the spread of artificial intelligence models, different implementations can support the planning process, such as the use of GANs. In this case, GANs can be exploited by adopting pre-existing location images resulting from experts’ judgments to illustrate the proposed intervention’s visual impact. To demonstrate the effectiveness of our hybrid model, we apply it to the city of Dublin. The results showcased how the method helps stakeholders, policymakers, and citizens in visualizing the proposed changes and gauging their potential impact with greater precision.

## Introduction

In recent years, with the rapid urbanization and the ever-increasing demands placed upon our cities, the importance of strategic spatial planning [13] emerged as a crucial component in shaping the sustainable futures of urban areas. As urban populations continue to swell, city planners and policymakers are confronted with the formidable challenge of mitigating the adverse environmental, social, and health impacts associated with urban sprawl, congestion, and pollution [23]. Spatial planning is inherently complex, reflecting the intricate interplay of diverse factors that influence the physical and social fabric of urban areas [19]. It demands an understanding of geography, demographics, economics, infrastructure, culture, and environmental considerations. The complexity arises from the need to harmonize competing interests, accommodate population growth, preserve natural resources, and enhance the quality of life for residents. Striking a balance between preserving historical heritage and pursuing modernization, or between urban density and open green spaces, illustrates just a fraction of the multifaceted challenges that planners face. Moreover, the dynamic nature of urban environments, subject to evolving technological, social, and economic trends, compounds the intricacy of spatial planning [3]. In navigating this complexity, urban planners must employ creativity, foresight, and adaptability to develop sustainable solutions that harmonize the diverse and often conflicting demands of urban living [21]. In this context, the concept of active transportation primarily advanced through the development of bike and pedestrian infrastructure, gains prominence as a multifaceted solution for addressing some of our era’s most pressing issues [1]. The provision of well-designed bike lanes, bike stations, and pedestrian zones within cities is of essential importance for several compelling reasons. The infrastructure investments promote active transportation, encouraging individuals to walk or cycle instead of relying exclusively on cars [25]. This shift not only reduces traffic congestion but also lowers the carbon footprint of urban areas, contributing to cleaner air and decreased greenhouse gas emissions [24]. Pedestrian and bike facilities enhance urban mobility and accessibility [9, 11], making it easier for people to access essential services, schools, workplaces, and recreational areas. This accessibility improves the overall quality of life for residents while reducing the need for car trips, which can alleviate traffic-related stress and improve urban air quality [2]. Furthermore, the establishment of secure and exclusive zones for pedestrians and cyclists not only promotes public health by stimulating physical activity and curbing sedentary habits but also lowers the likelihood of accidents (see among many [14]). This, in turn, contributes to creating more liveable and inclusive cities, catering to the needs of all residents, irrespective of their preferred mode of transportation. Nevertheless, locating suitable locations for these facilities can be a challenging endeavour for different reasons, including limited available space and the topography of a city, hills, rivers, and other natural features. In addition to physical constraints, urban planners must consider the needs and preferences of various stakeholders, such as residents, businesses, and local communities. In the scientific literature, different methods can be adopted to locate these facilities, including quantitative methods such as spatial models, GIS models, agent-based modelling etc. [9, 17, 18]. Nevertheless, quantitative methods are sometimes difficult to apply for two main reasons: (1) *Data availability*: they necessitate a significant volume of data, which is frequently either absent or only partially accessible for limited geographical regions [16]. (2) *Prediction*: these models offer a form of “forecasting” without considering the perspectives of experts, stakeholders, local and governmental authorities, or civilians [7]. For these reasons, a *mixed-methods* approach should be adopted in order to have a broader perspective of the study, considering both quantitative data and experts’ opinions.

In this study, we consider the use of the Real-time spatial Delphi (RTSD) [8, 12], a spatial version of the Delphi method [20], particularly useful for facilitating a spatial consensus among a panel of experts adopting a real-time virtual environment, statistical algorithms and spatial and textual analysis. Specifically, for this paper, we adopt “Real-Time Geo-Spatial Consensus System” (www.rtgscs.com–v.3.0) [8], a novel web-based open platform, specifically useful to obtain convergence of opinions among panellists in a short time. Real-Time Spatial Delphi is particularly useful in uncertain conditions when there is a lack of quantitative data and/or when experts’ judgments are requested in order to have a spatial perspective ready for policy implementation. Nevertheless, the outputs of RTSD are spatial representations in the form of maps, not immediately visible in reality. With the proliferation of AI models, diverse applications can bolster the planning procedure, including the utilization of Generative Adversarial Networks (GANs). In this context, GAN models [10] can be adopted after the iterative decision-making process by employing existing images of potential intervention sites (such as bike lanes, bike stations and pedestrian zones) to generate novel visuals illustrating the envisioned modifications. The proposed hybrid approach offers several advantages over traditional planning methods. Traditional methods such as GIS models, agent-based modelling, and spatial analysis rely heavily on extensive datasets, which are often incomplete or unavailable for many regions (as in our case). Moreover, these methods tend to focus on the prediction of the outcomes without adequately incorporating expert opinions, leading to potential biases. In contrast, our Real-Time Spatial Delphi method, integrated with Generative Adversarial Networks, combines expert knowledge with data-driven insights, providing a more holistic and flexible approach to urban planning. This hybrid method allows for real-time adjustments based on expert feedback, enhancing the accuracy and relevance of the planning outcomes. In sum, this methodology aids stakeholders, decision-makers, and citizens in conceptualizing the proposed images and conducting a more precise assessment of their potential ramifications. To demonstrate the effectiveness of our hybrid model, we apply it in the context of the city of Dublin. Overall, the research objectives of this paper are the following:

1. ${RO}_{1}$: Propose a novel method for participatory planning, combining Real-Time Spatial Delphi to assign priorities and Generative Adversarial Networks to visualise potential policies in reality.
2. ${RO}_{2}$: Facilitate the efforts of policymakers by providing a visual representation of the policies, ready for evaluation.
3. ${RO}_{3}$: Apply the method to active mobility infrastructures in the city of Dublin.

The remainder of the paper is organized as follows. This section serves as an introduction, presenting the background of the study, the related literature, and the research objectives. Section 2 outlines the methods employed in this study and the combination of Real-Time Spatial Delphi and Artificial Intelligence. Section 3 emphasizes the results achieved, through spatial statistics, statistical indicators, and advanced analysis. Ultimately, in Sect. 4, we provide concluding remarks while considering possible directions for enhancing this model in future research.

## Methods

**Fig. 1:**
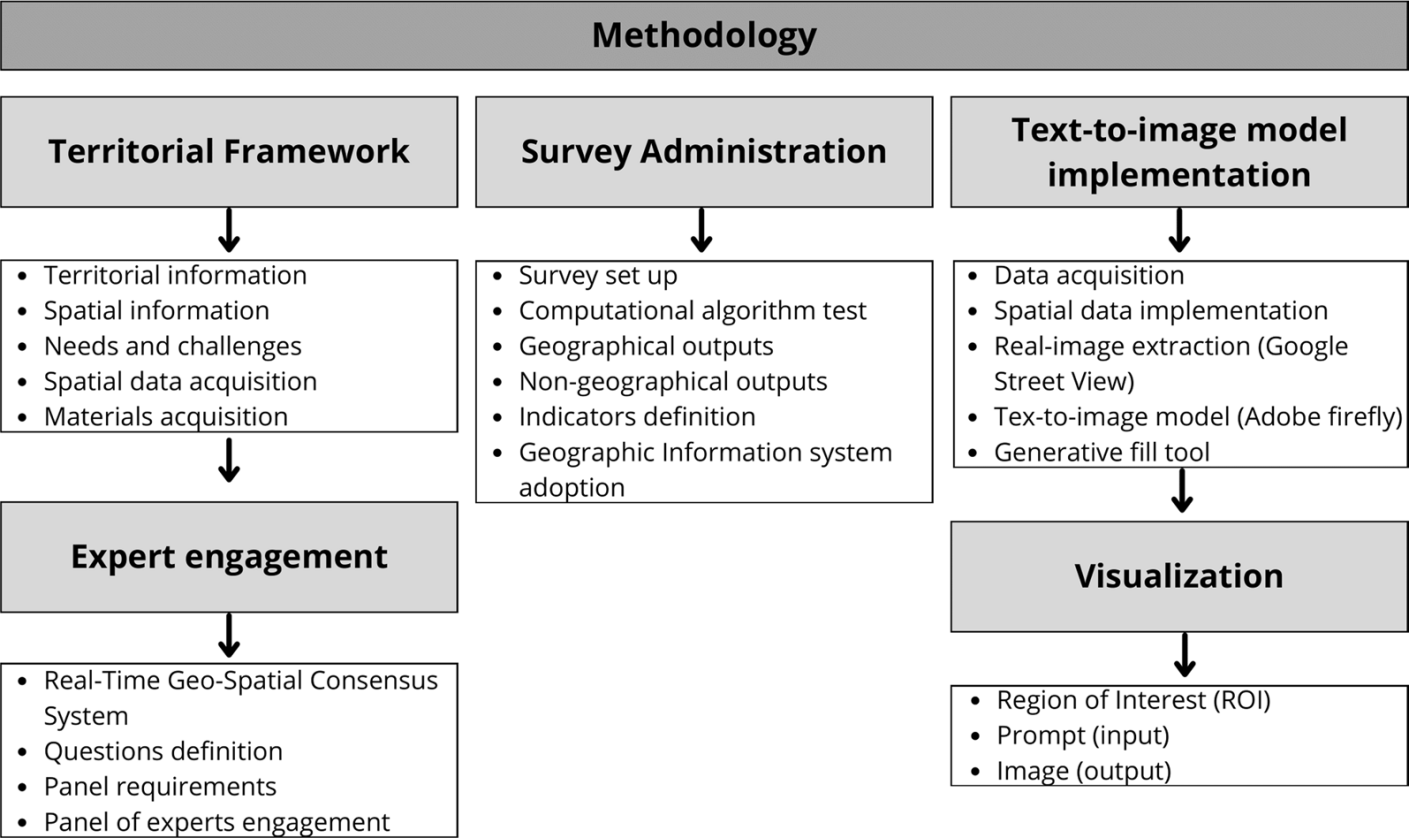
Proposed methodology

In this section, an illustration of the hybrid method adopted in this paper is provided. We follow a revised version of the approach proposed by Bishop et al. [4] and Calleo et al. [8] in the Futures Studies context, implementing a novel phase employing the use of Artificial Intelligence. The method proposed in this paper follows a specific research design and is composed of three main stages (see Fig. 1). In sub Sect. 2.1, we present the territorial framework, examining cycling facilities in Dublin City. From this exploration, we formulate research questions to present to our panel of experts. Moving on to sub Sect. 2.2, we detail the method, showing computational and statistical algorithms utilized by the system, along with its real-time analysis, chosen indicators, and the examination of outputs. Lastly, in sub Sect. 2.3, we explain how the survey results are considered in the text-to-image (T2I) model, generating visual representations of the insights gleaned from expert opinions.

### Territorial framework and experts’ engagement

In this phase, we outline the main process of our survey, defining different variables as they may affect the final results. Firstly, we conduct a specific analysis of the territorial framework, regarding our case study, i.e., the city of Dublin, the specific needs regarding urban planning, and the specific requests for bike lanes, bike stations and pedestrian zones for our interests. Dublin is a reasonably bicycle-friendly city [15], there are a variety of dedicated bike lanes and routes throughout the city,Dublin boasts several bike-sharing initiatives (Dublinbikes, Moby bikes and Bleeper), offering the convenience of renting a bicycle for a modest fee and returning it at numerous bike stations dispersed throughout the city. Overall, while there are certainly some challenges to cycling in Dublin (such as heavy traffic in some areas), the city has made significant efforts in recent years to improve its infrastructure and support for cyclists. Nevertheless, different problems emerged such as the lack of specific barriers and suitable locations for pedestrian zones within the city of Dublin. In particular, their distribution is constrained, with a predominant concentration in the central part of the south of the city (close to the Temple Bar area).

Following the Delphi method [5], after determining the territorial framework, we acquire possible materials available, both from the literature and from spatial data in order to give the experts a perspective of the study. This is important to provide essential elements of the study and existing literature reducing the drop-out rates. Specifically, we conduct an analysis of spatial data sourced from the Dublin City Council, focusing on existing facilities (bike lanes, bike stations and pedestrian lanes). This analysis aims to provide experts with insights into the overall territorial context in order to avoid choices where facilities are already implemented. The spatial results, along with additional materials such as existing reports, recent implementations, and technical guides on how to use the platform, are uploaded directly into the platform ready for the administration of the survey. In particular, we incorporated maps for the three facilities using open data supplied by the Dublin City Council for bike lanes and bike stands. Regarding pedestrian zones, due to the limited coverage, we created a visual map pinpointing these specific areas. This information proves invaluable to experts as it offers a comprehensive overview of the existing facilities.

In this study, we adopt “Real-Time Geo-Spatial Consensus System (www.rtgscs.com-v.3.0) [8], a novel platform exploiting real-time collaboration of experts, adopting GIS tools, spatial and textual analysis in order to obtain a convergence of opinions on the territory. At this stage, we define the research questions ($RQ$) to pose to the experts, and since we want to investigate proper locations for bike lanes, bike stations and pedestrian zones, we ask the following questions to a panel of experts:

1. ${RQ}_{1}:$ Please identify on the map suitable locations for bike lanes.
2. ${RQ}_{2}:$ Please identify on the map suitable locations for bike stations.
3. ${RQ}_{3}:$ Please identify on the map suitable locations for pedestrian zones.
  These general questions are well organised into the platform, attached with the geographic location expressed in $x,y$ points, where experts can provide judgments directly by placing one or more points and textual comments to justify their answers. For this study, we engage a panel of experts with high levels of expertise in the following areas: transport planning, urban design, traffic engineering, accessibility, GIS and data analysis, project management and technology and innovation. More in detail, we invited 40 selected experts ($E$) and among them, $E= 15$ participated in our study (Table 1), with an average acceptance rate of 37.5% to our invitation. In particular, the panel was composed of three main categories, including academic people (11 experts), local authorities (3 experts), and a member of a transportation company (1 expert), thus allowing us to acquire a broader perspective of the research area.

**Table 1:** Panel of experts

Contacted experts	Number	Participating experts	Number
Academic	25	Academic	11
Local authorities	10	Local authorities	3
Company	5	Company	1
Total	40	Total	15

The three primary categories are evaluated by examining the key transport decision-making bodies in Dublin, assessing territorial policies, recent publications, and current projects [15, 26]. However, in the traditional Delphi method–and in decision-making processes where subjective judgments are involved—pinpointing a representative or probabilistic expert sample proves challenging. To overcome this challenge, the panel of experts must encompass a wide range of diversity in terms of expertise to ensure a rich array of skills and perspectives on the subject matter [7].

### Survey administration

In this case, we enrol experts on the platform by using their email addresses as credentials. Once they log in to the RT-GSCS, they are presented with a primary interface featuring an interactive map. From a sidebar, they can choose questions and respond in real time by marking one or more points on the map. Once they mark a location, an automatic circle appears, moving, shrinking, and expanding in real-time based on the anonymous responses from other experts. The experts have the option to justify their judgments at any time by providing comments or feedback. The statistical algorithm implemented in the platform is suggested by Di Zio and Pacinelli (2011) and it is performed in real-time with the aim of obtaining a spatial consensus on the territory. To pursue this objective, RTSD employs a computation algorithm performed in real-time integrated in the main system. From the first point acquired, a set of $N$ judgments are considered ($N={\sum }_{e=1}^{E}{N}_{e}$), and for each question, a geometric element identified as a circle $C$ is displayed, representing the spatial consensus on the territory. The algorithm, similar to the $IQR$ range adopted in the traditional Delphi method, find a circle $C$, the smallest one among the possible ${C}_{i}$, containing half of the $N$ opinion points. Following this aspect, a representation of the $IQR$ is performed in real-time on the dynamic map with the circle $C$, including 50% of the $N$ judgments with the attached $x,y$ coordinates. This approach is adopted because a mere clustering of experts’ responses may not suffice in reaching a consensus. Additionally, certain opinion points might be situated in illogical or incorrect geographical areas, potentially altering the final results.

During the survey, if the experts answer to the different questions proposed, we obtain a spatial distribution of $N={n}_{1},{n}_{2},\dots , {n}_{N}$ points on the map for each question. In this case, for each question, the algorithm finds a minimum area ${A}_{i}$ of the circle ${C}_{i}$, covering 50% of the points, denoted as:

1 $$A_{i} \supseteq T_{{\left( {N/2} \right)}}$$

where ${T}_{N/2}$ is the 50% of the $N$ points. Nevertheless, one of the main issues to consider is the existence of an infinite array of possible circle computations and for this reason, to determine spatial consensus on the map, we consider that ${C}_{i}$ must have the centre in one of the $N$ points. This choice stems from the observation that in the conventional Delphi method, judgments are most of the time analysed by considering $IQR$ outputs and assessing potential outliers, all in pursuit of attaining consensus.

From this premise, the algorithm performs a calculation for each question of a minimum area $min(A)$, denoted as $min(A)=Med({\varvec{A}})$, where for each question, the vector ${\varvec{A}}=[{A}_{1},{A}_{2},\dots {A}_{N}]$, is obtained adopting a matrix $M$, with all the distances between $N$ points. The matrix is composed of $N$ columns and $N$ rows, and each intersection value represents the straight-line distance between the points. The median ($Med$) of the ordered vector ${\varvec{A}}$ corresponds to the area ${A}_{i}$ centred on pint ${n}_{i}$ containing 50% of the $N$ points. Finally, $min(A)$ denotes the spatial consensus on the territory [6]. With this approach, we have two types of final outcomes derived from the survey: (1) *Geographical results*: the judgments represented in an interactive map, including the distribution of $N$ points and the circle of convergence $C$. (2) *Non-geographical results*: the information obtained from the dynamic process, including spatial data (e.g., coordinates, polygons, informative points, clusters), spatial measurement (including the circle size over the time) and textual comments provided by the experts. In our case, textual comments are important to understand the cooperation among the panel, specifically to understand the policies suggested by the experts in specific locations. Based on this premise, while geographical results provide immediate visualization, they do not portray the precise process of convergence, and for these reasons, we adopt three main statistical indicators: ${M}_{1},{M}_{2}, {M}_{3}$ [8, 12]:

2 $$M_{1} = FC\left( {km^{2} } \right)$$

where $FC$ represents the final circle area expressed in ${km}^{2}.$ This metric is useful for the identification of the portion of the territory and the relative circle size. Nevertheless, this measurement is absolute and does not consider the study area boundaries or the size of the initial circle. To address this challenge, we also consider a second indicator:

3 $$M_{2} = 1 - \frac{FC}{S}$$

calculated as the ratio between the final circle’s area ($FC$) and the surface ($S$) of Dublin ($S=117.8 {km}^{2}$). This indicator illustrates the degree of geo-consensus, and the closer the measure is to 1, the smaller the consensus circle is in relation to the surface. Finally, the third indicator measures the dynamic process of spatial convergence:

4 $$M_{3} = \frac{FC}{{IC}} - 100$$

where $IC$ is the initial circle area, and the higher the value (closer to 100%), the poorer the convergence of opinions; conversely, the closer it is to zero, the stronger the convergence (we refer to $IC$ as the circle derived from the second point expressed by the expert, as the first absolute one is preset as 50 ${km}^{2}$ and cannot be included in the interquartile calculation).

In our survey, for each opinion-point ($${n}_{i})$$, we provide experts with the option to assign weight, on a Likert scale ($$w=1-5$$), indicating the level of priority within the territory. This functionality is particularly beneficial for later analyses, including the identification of hotspots or clusters of points with significant importance. In this study, we employ ArcGIS PRO for the analysis of spatial data, performing a heatmap analysis that highlights potential hotspots within the area, considering the expert-provided weights.

As explicitly mentioned earlier, our process takes place in a real-time virtual environment, so a criterion for ending the survey must be established. In the literature, there is no consensus regarding the criterion determining the survey’s conclusion. Especially in this context, it could be considered that the convergence of opinions serves as a valid criterion for concluding the exercise. However, von der Gracht (2012) asserts that more than convergence, it is important to consider stability over time. Therefore, we proceed to perform a time-series analysis and conclude the exercise when there is no significant variation (defined here as 5% of the $N$ points) in the overall distribution of points.

### Text-to-image model implementation

**Fig. 2:**
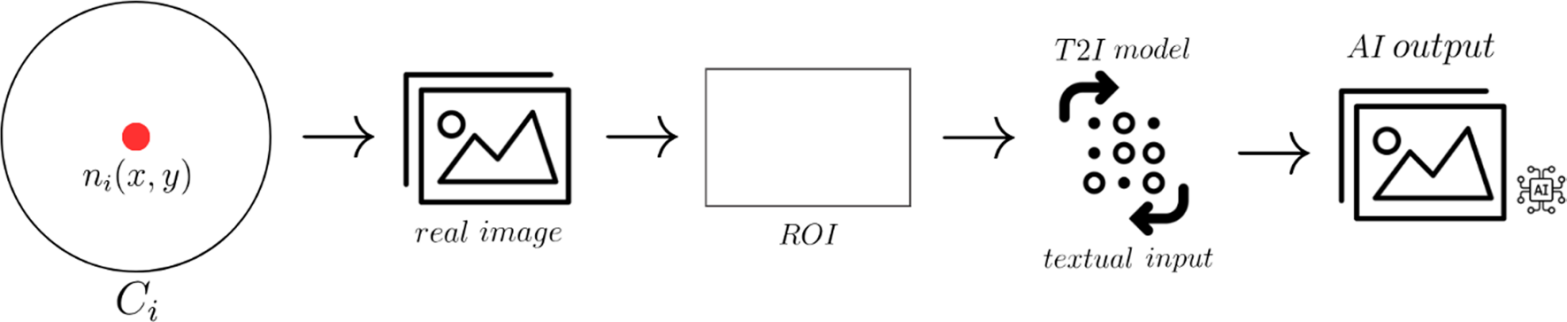
Adopted model

Upon concluding the survey and the iterative process, we obtain the final results, ready to be integrated into the text-to-image model for visualizing the real-world implementation of the proposed facilities. Specifically, if we obtain a total distribution of $N$ points for each question, we consider the last ${n}_{i}$ point inside the circle ${C}_{i}$, and we extract the real image. In this case, we extract the last ${n}_{i}$ point to showcase our method, however, the approach is flexible, and it can be proposed for the total distribution of $N$ points. Specifically, the coordinates ($x,y$) of the ${n}_{i}$ are considered and imported into a tool that enables us to visualise the real image of the area. In this case, we use Google Street View in order to extract the specific image of the street of correspondence. This output is subsequently imported into a text-to-image model, in this case Adobe Firefly (*Beta*) in order to generate a novel image with the facility implemented. An overview of the method is illustrated below (Fig. 2).

T2I models are extremely useful for generating new images starting from a textual input, with the use of Generative Adversarial Networks. GANs are central to text-to-image models. These unsupervised machine learning systems are built to produce synthetic data that mimics a given training dataset. Rather than using labelled data, GANs learn to differentiate between real and synthetic data through a competitive process. In this case, GANs consist of two main neural networks, a generator $G$ and a discriminator $D$, considering two main variables, real and fake images. In fact, the generator creates from a textual input novel rendering, while the discriminator tries to understand how to differentiate these outputs, based on a correlation. It is important to emphasize that, in contrast to conventional assumptions, the primary task of the generator is not to predict the label with the attached details but instead to predict the details of the hypothetical label. In this term, we define as a two-player minmax game the process, where $G$ is revised according to the $D$ network’s performance, while $D$, in turn, receives updates based on the performance of the generator network. In this process, an “adversarial” dynamic is processed, where the generator tries to “fool” the discriminator, and the discriminator tries to “catch” the generator [22]. Technically, if we consider the min–max game denoted as ${min}_{G}{max}_{D}V\left(D,G\right)$ between the generator network $G$ and the discriminator network $D$, where the objective function is represented by $V(D,G)$, we aim to minimize $V(D,G)$ with respect to $G$ and maximize it with respect to $D$. This function, $V(D,G)$ can be expressed as an expectation over a probability distribution. Specifically, let ${P}_{data}(x)$ denote the distribution of real data samples and ${P}_{G}(z)$ represent the distribution of synthetic data samples produced by the generator. For a given real sample $x$, the $log$ probability that the discriminator correctly identifies it as real is $logD\left(x\right)$. Conversely, the log probability that discriminator mistakenly classifies a synthetic data sample $G(z)$ as real is $log(1-D\left(G\left(z\right)\right)$. Based on this assumption we obtain the following formula:

5 $$\min_{G} \max_{D} V\left( {D,G} \right) = {\mathbb{E}}_{x } \sim P_{data} \left( x \right) \left[ {\log D\left( x \right))} \right] + {\mathbb{E}}_{z } \sim P_{data} \left( z \right)\left[ {\log \left( {1 - D\left( {G\left( z \right)} \right)} \right)} \right]$$

Generally, one common method for training a GAN involves using the binary cross-entropy loss function. This approach measures the discrepancy between the predicted probabilities output by the discriminator and the true labels (1 for real images and 0 for synthetic images). In this setup, the generator’s goal is to minimize this loss by creating synthetic images that are more likely to be classified as real.

**Fig. 3:**
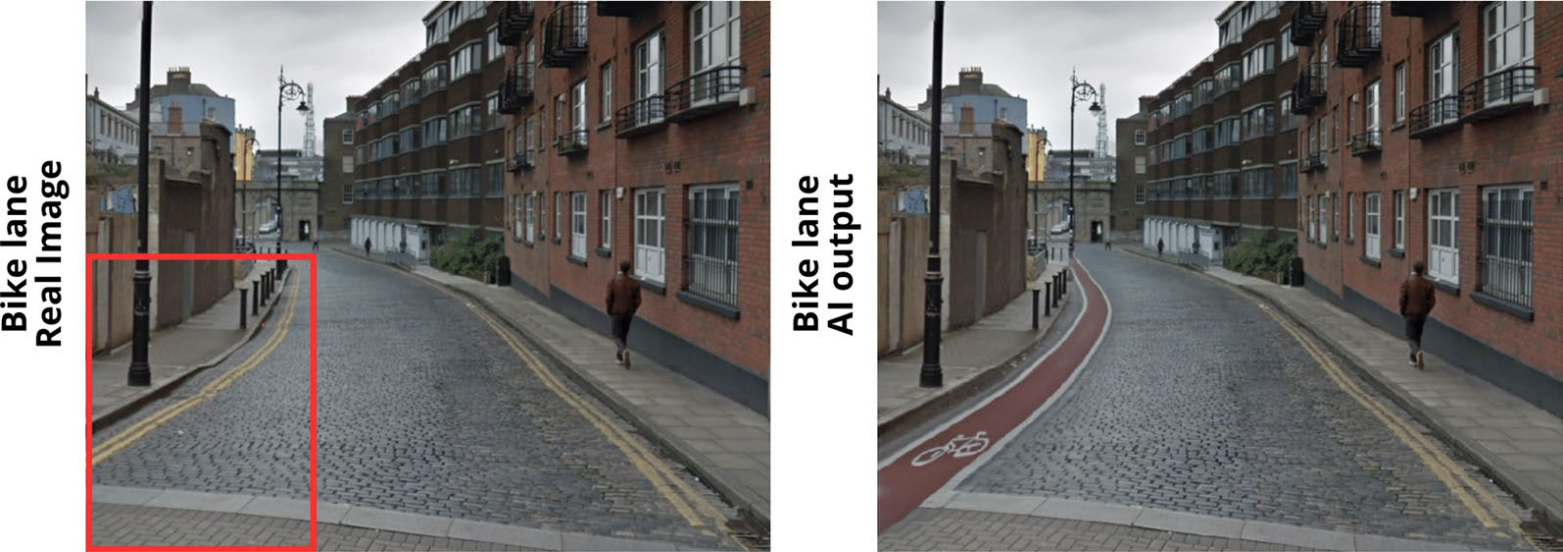
Model implementation example

At this stage, we import the extracted real image for each research area into the model of Adobe Firefly, adopting the “Generative fill” tool. Once the image is selected and imported, we move forward with the identification of a Region of Interest (ROI), which denotes particular image areas where the AI undertakes the generation process based on the provided text input. This step holds great significance as it enables the model to focus exclusively on the desired area of interest. After identifying the ROI, we employed a textual prompt to generate the novel images. Specifically, for bike lanes: “*Generate a bike lane*”, for bike stations: “*Generate a bike stations*”, for pedestrian zones: “Generate a pedestrian zone”. The novel images are then used to be of support for communication and dissemination, furthermore, they could be used for assessment by the experts. An example of the model process is depicted in Fig. 3.

## Results and discussion

In this section, we emphasize the ultimate findings of the application of the method; in particular, in sub Sect. 3.1, we depict the spatial results mapping the encompassing $N$ judgments and the consensus circle. Furthermore, a heat map analysis is performed to outline possible hotspots and clusters on the territory. Subsequently, in sub Sect. 3.2, we analyse the spatial measures and further data obtained from the study through the statistical indicators and time series. Finally, in sub Sect. 3.3, the results from the combination of Real-Time Spatial Delphi and the text-to-image model are presented, providing a visual representation of the suggested policy.

### Spatial analysis outputs

**Fig. 4:**
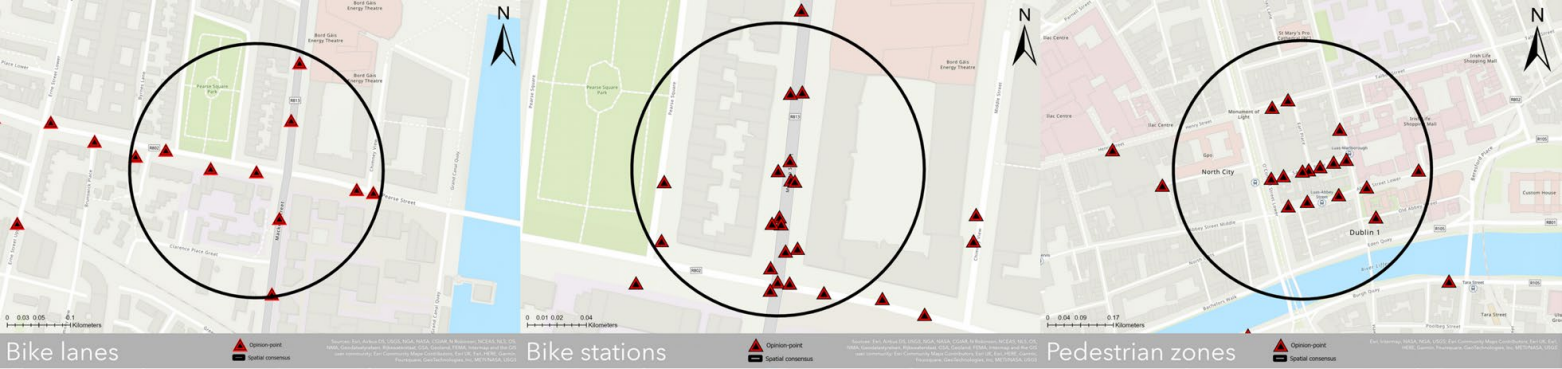
Spatial results analysis

After gathering the spatial outcomes from the collaboration of the expert panel, we imported them into ArcGIS PRO, generating three primary maps (Fig. 4). The maps depict the outcomes derived from the survey session in the identification of pertinent roads and areas for the three main questions and are representative of the distribution of $N$ points with the final consensus circle $C$. Specifically, for ${RQ}_{1}$, in the identification of suitable bike lanes, experts have pinpointed a high-priority location situated in the eastern part of the city centre, in close proximity to Macken Street. This particular area holds significant importance according to these experts, as it serves as a crucial linkage point between the Docklands area and Dublin 4, intersecting with Pearse Street. Alongside inputting spatial points, the experts also provided insightful comments during the analysis phase, enhancing the understanding of geographical aspects. Based on the experts’ comments this area is characterized by heavy traffic congestion, particularly during peak hours, and witnesses a substantial volume of cyclists and e-scooter riders. This surge in non-motorized transportation is largely attributable to the nature of Grand Canal, which runs through the vicinity. With regards to ${RQ}_{2}$, experts have identified suitable locations for bike stations in the eastern section of Dublin, near the Grand Canal, where they successfully achieved a convergence by adding multiple points. Based on their judgments, these strategically located bike stations hold significant value for individuals, as they are in proximity to various amenities such as shops, gyms, and markets. The addition of more advanced and innovative bike stations for personal bicycles in this area would prove highly beneficial. Finally, for ${RQ}_{3}$ pedestrian zones are primarily situated in the city centre, mainly for tourism-related purposes. Experts suggest that the ideal area for pedestrian zones can be found around the Grand Canal, specifically in some side streets branching off from O’Connell Street.

**Fig. 5:**
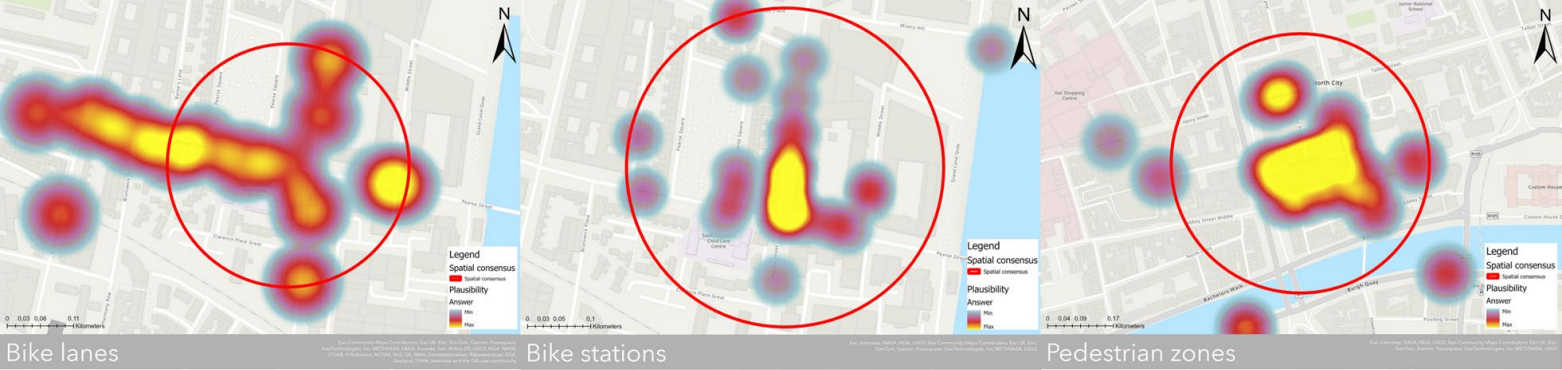
Heatmap analysis

After obtaining a spatial representation of the results, depicting the convergence of opinions across the surveyed territory, we proceed with a heatmap analysis (Fig. 5) to pinpoint clusters of data points associated with the expert-assigned weights. Indeed, experts were given the opportunity to allocate a weight, denoted as $w$, to each inserted point using a Likert scale, facilitating the assessment of priority across the territory. In this case, the consensus circle is derived from the ${M}_{1}$ indicator and the heatmap is performed adopting the $w$ assigned by the experts as a weighted field and a variable radius for the three maps (for ${RQ}_{1}$, $r=45m$, for ${RQ}_{2}$, $r=30m$, and for ${RQ}_{3}$, $r=35m$) based on the proximity of the points.

The findings are noteworthy, revealing a strong emphasis on the top three facilities. In fact, the majority of expert assessments, rated on a scale of $w=1-5$, predominantly fall within the 4 and 5 range. An interesting aspect is the alignment between bike station placements and bike lanes, with the identified area showing high proximity in terms of priority. This underscores the importance of ensuring the close proximity of both facilities. Additionally, our findings reveal a noticeable trend where higher values tend to concentrate within the geographical consensus circle. This observation should not be taken as implying a clear cause-and-effect relationship between the geo-consensus circle we have defined and the high values it contains. This assumption does not universally apply for several reasons. Firstly, due to the real-time nature of the described process, experts have the flexibility to input one or more points on the map and assign them a plausible weight. Particularly during the initial stages, the circle undergoes frequent adjustments, expanding or reducing in size. Moreover, a point input by an expert with a high weight at a specific $x,y$ coordinates may initially fall within the circle but later end up outside of it. This highlights the key point that experts may not necessarily be influenced by the points residing within the circle when assigning weights to their designated points, whether those points are inside or outside the circle. Furthermore, it underscores that a direct correlation between the high plausibility assigned to these points and the final configuration of the circle cannot always be definitively established.

### Dynamic process of convergence

In this paragraph, we delve further into the dynamic convergence process resulting from expert collaboration. Table 2 provides an overview of key information, including the research area, which covers 117.8 ${km}^{2}$, the initial circle ($IC$), the final circle ($FC$) expressed with the indicator ${M}_{1}$, along with the indicators ${M}_{2}$ and ${M}_{3}$. Finally, the distribution of $N$ points, with the relative comments are indicated.

**Table 2:** Spatial measurements

Research area	Study area (${km}_{2}$)	Initial circle (${km}_{2}$)	Final circle (${km}_{2}$)${M}_{1}$	${M}_{2}$	${M}_{3}$(%)	Points ($N$)	Comments
Bike lanes	117.8	2.15	0.12	0.998	5.58	21	7
Bike stations	117.8	1.92	0.15	0.998	7.81	30	5
Pedestrian areas	117.8	2.25	0.20	0.998	8.88	18	6

With respect to ${RQ}_{1}:$ “*Please identify on the map suitable locations for bike lanes*”, experts placed 21 opinion-points, along with 7 useful comments. In this question a significant reduction of the initial circle size occurred, decreasing from an $IC=2.15$ to a final circle size $FC=0.12$, ultimately leading to a successful consensus. The second indicator ${M}_{2}$, demonstrates a convergence of opinions achieved on the territory, in fact, the value is close to 1, with ${M}_{2}=0.998$. Finally, to understand better the dynamic process, we highlight the result from the metric ${M}_{3}=5.58$. The results from this indicator demonstrate a strong convergence of opinions, and we can consider achieved the convergence because ≤ 20% (von der Gracht, 2012). For ${RQ}_{2}$: “*Please identify on the map suitable locations for bike stations*”, we have a total distribution of $N=30$ points and 5 comments along with a reduction $IC$, where $FC=0.15$. In this case, the spatial consensus is obtained since we have a value close to 1, equal to ${M}_{2}=0.998$. Furthermore, the results from the indicator ${M}_{3}=7.81$ are interesting and demonstrate the achieved convergence among the panellists. Finally, with regards to ${RQ}_{3}$: “*Please identify on the map suitable locations for pedestrian zones*”, the resulted opinion-points are $N=18$, with 6 comments. Also in this case, we obtained a strong reduction of $IC$, with $FC=0.20$. Moreover, we can consider achieved a substantial convergence of opinions for the last two indicators, where ${M}_{2}=0.998$ and ${M}_{3}=8.88$.

**Fig. 6:**
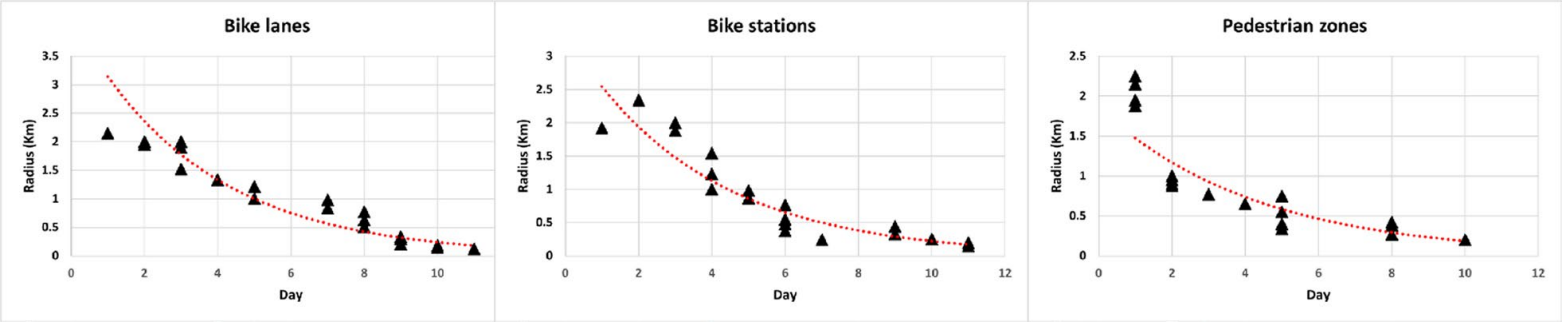
Time series

After presenting the spatial measurements, we conduct a time-series analysis to gain a deeper understanding of the process’s evolution over time and to conclude the survey (Fig. 6). In our case, for all the three research questions, we have a substantial reduction of the circle size over time, and we acquire a convergence of opinions and a stability in less than 12 days. In particular, for ${RQ}_{1}$, we observe a fluctuating situation during the initial five days, followed by a gradual reduction, ultimately stabilizing by the 11th day. This is an extremely interesting results, since in the traditional version of the Delphi method, long time frames should be considered (typically around 2–3 months). Specifically, the daily average of points entered per day was almost 2 points fluctuating on the third and fifth day and on the eighth and ninth day. For ${RQ}_{2}$, the circle size changed at the beginning of the exercise, decreasing from the 4th day, and reaching a consensus on the 11th day. Here, we have an average of more than 2 points per day, with a non-fluctuating trend but with an exponential increase on the second and sixth days, with 4 and 5 points entered respectively. Finally, for ${RQ}_{3}$, the experts reached a convergence of opinions in less time, and from the 2nd day, we can see a reduction of the circle, acquiring a stability on the 10th day. In this question, an average of almost 2 points were entered on the indicated days, with a higher insertion rate on the first and second days with 4 points, and on the fifth and eighth days with 4 and 3 points respectively. Overall, from the trend line of the three research area we can note a substantial reduction, that could be analysed in future works with more robust statistical test to approximately understand the time frame of a Real-Time Spatial Delphi survey.

### Visualization

**Fig. 7:**
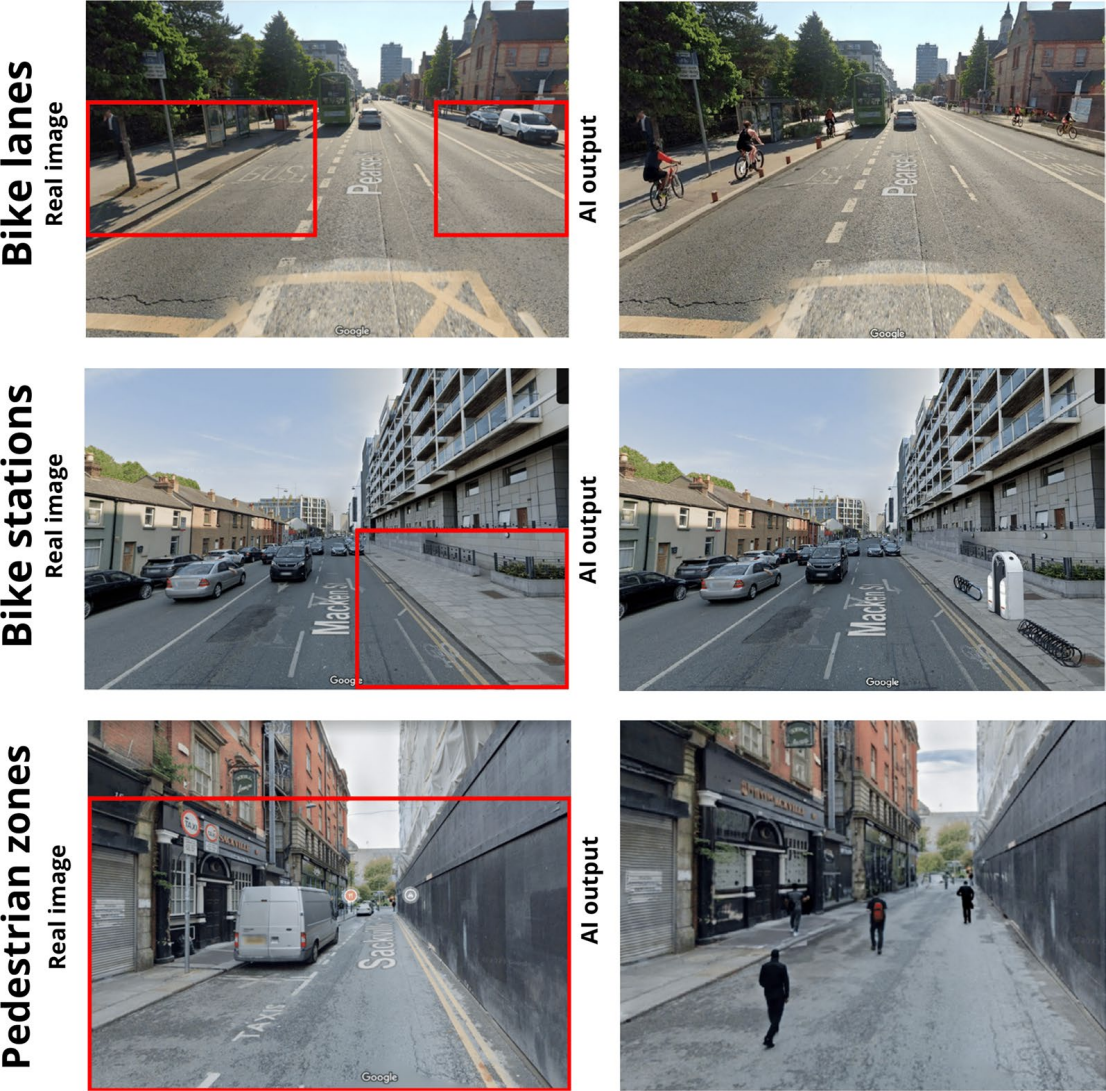
AI outputs

Once we obtain the final results and the iterative process is concluded, we can generate a visual representation of the suitable locations identified by the experts (Fig. 7) adopting T2I models. In spatial planning and policy development, the visual aspect is extremely important, for two main reasons: (1) *Policy assessment*: a visual representation of spatial points, can help policymakers better understand the data, as visualizations can be more intuitive and easier to interpret than raw data or text descriptions alone. Furthermore, allows a quick identification of patterns, trends, and anomalies in the spatial data, with comparative analysis ready for decisions. (2) *Communication*: visual representations are often more accessible and easier to understand than raw data or complex textual descriptions. By using images, policymakers can convey information in a clear and straightforward manner, making it more comprehensible to a wider audience, including those with varying levels of expertise. For these reasons, we present in this paragraph, a visualisation of the facilities implemented in the territory suggested by the experts. To showcase our method, we consider the last point ${n}_{i}$ within the circle ${C}_{i}$, with coordinates $x,y$. The results are really interesting and propose for the first time, a visual representation derived from the Real-Time Spatial Delphi survey, but overall, from a decision-making process. This method is useful to acquire spatial points with the suggested policies (in this case the logistic facilities) and represent reality. The clear visualisation of the implementation is useful for experts, decision-makers and policy makers in order to visualise with low effort the proposed policies ready for evaluation. Nonetheless, it is essential to emphasize that this approach does not provide a readily implementable visual representation replacing human decisions. Nevertheless, it serves as a valuable support method for evaluation and communication purposes. In Fig. 7, we can observe the three main facilities implemented within the territory using T2I models.

On the left, a real image of the territory is displayed, including the Region of Interest, while on the right, we showcase the generated model output. For ${RQ}_{1}$, we can emphasize how the model produced an image featuring the incorporation of a bike lane on both the left and right sides of the street. In this case, it is interesting to note how the model implements also the cyclists associating the lane with people riding their bikes. For ${RQ}_{2}$, we provided the model with a prompt to generate bike stations, and we specify the ROI on the sidewalk. In this scenario, the model effectively generated bike racks that incorporate charging stations for electric bikes (at least from an ideal perspective). Finally, for ${RQ}_{3}$, we have expanded the size of the ROI as we aim to generate the entire street as a pedestrian zone. In fact, the output is a visualisation of the street as a pedestrian zone with pedestrian walking.

Based on the findings, the experts pinpointed areas of high priority for implementing the examined facilities and offered various recommendations. This method thus furnishes a crucial tool for decision-making bodies, allowing them to swiftly and clearly visualize the “reality” of implemented policies once the results of the convergence process are obtained. Beyond the methodological aspect, the application aspect also offered essential and insights into potential locations for cycle paths, a much-debated topic in Dublin’s territorial planning, as well as pedestrian areas and bike stations. It is important to emphasize that this approach should be viewed as complementary to expert opinion, and the images provided should not be deemed definitive but rather as aids for real planning or dissemination purposes.

## Concluding remarks and future works

In this study, we have explored the intricate field of urban spatial planning, acknowledging its crucial role in shaping sustainable urban futures. As cities face challenges such as sprawl, congestion, pollution, and the need for improved mobility, our research aims to provide innovative solutions through a novel integration of Real-Time Spatial Delphi and Generative Adversarial Networks. We have successfully met our research objectives by proposing a method that combines RTSD and GANs, offering a powerful tool for visualizing potential urban policies. Our approach exploits the collective wisdom of experts and generates visual representations to help decision-makers and stakeholders gain a more comprehensive understanding of proposed urban interventions compared to traditional methods such as GIS modelling, agent-based modelling, and spatial forecasting. By applying this method to the city of Dublin, we have demonstrated its practicality and effectiveness in a real-world context. However, there are some limitations to consider. The generated images have constraints related to planimetry and may not account for specific urban regulations. Additionally, subjectivity in the implementations may affect their utility. Despite these limitations, the visualizations can serve as valuable initial resources for policymakers, streamlining the evaluation and implementation of urban policies related to bike lanes, bike stations, and pedestrian zones. The proposed hybrid method represents a significant advancement in urban planning. Compared to traditional methods, it offers greater flexibility and real-time adaptability, allowing for improved management of uncertainties and limitations in quantitative data. This method enhances the accuracy of participatory approaches and makes the planning process more transparent by directly involving experts and employing realistic visualizations of potential urban modifications. Nevertheless, certain challenges persist, including data availability, which is a major concern because the quality and completeness of input data significantly influence the outcomes, as well as ensuring model accuracy and generalizability to other cities, which is challenging due to specific urban contexts and local planning regulations. Indeed, low response rates from categories such as local authorities and transport companies highlight the difficulty in engaging a well-structured panel of experts, who may be too occupied to participate in surveys. Future research could address these challenges and explore several avenues. While our study focused on bike infrastructure and pedestrian zones in Dublin, the method could be extended to other urban interventions, including public transportation, green spaces, and mixed-use developments. Future studies could also integrate various geometric shapes relevant to the subject of investigation, such as ellipses, triangles, and lines, to enhance convergence on these aspects. Incorporating multiple circles could improve the procedure by accounting for distinct clusters within the territory. Additionally, future research should integrate environmental and social factors—such as air quality, noise pollution, and social equity—to provide a more holistic perspective. Refining GAN models to enhance the realism and accuracy of generated images is also crucial. Comparative studies with traditional planning methods can offer further insights into the strengths and limitations of our approach. Developing automated tools to streamline the integration of GANs into urban planning platforms can make the process more efficient and accessible for policymakers. In conclusion, the integration of Real-Time Spatial Delphi and Generative Adversarial Networks offers a promising pathway for transforming urban planning into a more transparent, data-driven, and participatory process for both policymakers and citizens. Future research should continue to refine these methods and explore their application across diverse urban contexts to fully realize their potential.

## Data Availability

Data are available on request.
